# Impact of excessive alcohol abuse on age prediction using the VISAGE enhanced tool for epigenetic age estimation in blood

**DOI:** 10.1007/s00414-021-02665-1

**Published:** 2021-08-18

**Authors:** Danuta Piniewska-Róg, Antonia Heidegger, Ewelina Pośpiech, Catarina Xavier, Aleksandra Pisarek, Agata Jarosz, Anna Woźniak, Marta Wojtas, Christopher Phillips, Manfred Kayser, Walther Parson, Wojciech Branicki, the VISAGE Consortium

**Affiliations:** 1 Jagiellonian University Medical College, Faculty of Medicine, Department of Forensic Medicine, Grzegórzecka 16, 31-531, Krakow, Poland; 2 Institute of Legal Medicine, Medical University of Innsbruck, Muellerstrasse 44, 6020 Innsbruck, Austria; 3 Malopolska Centre of Biotechnology, Jagiellonian University, Gronostajowa 7A, 30-348 Krakow, Poland; 4 Central Forensic Laboratory of the Police, Aleje Ujazdowskie 7, 00-583 Warsaw, Poland; 5 Forensic Genetics Unit, Institute of Forensic Sciences, University of Santiago de Compostela, R/ San Francisco s/n, 15782 Santiago de Compostela, Spain; 6 Department of Genetic Identification, Erasmus MC University Medical Center Rotterdam, PO Box 2040, 3000 CA Rotterdam, The Netherlands; 7 Forensic Science Program, The Pennsylvania State University, 13 Thomas Building, University Park, PA 16802 USA

**Keywords:** DNA methylation, Alcohol abuse, Epigenetic age prediction, VISAGE enhanced tool for age estimation of DNA from somatic tissues

## Abstract

DNA methylation-based clocks provide the most accurate age estimates with practical implications for clinical and forensic genetics. However, the effects of external factors that may influence the estimates are poorly studied. Here, we evaluated the effect of alcohol consumption on epigenetic age prediction in a cohort of extreme alcohol abusers. Blood samples from deceased alcohol abusers and age- and sex-matched controls were analyzed using the VISAGE enhanced tool for age prediction from somatic tissues that enables examination of 44 CpGs within eight age markers. Significantly altered DNA methylation was recorded for alcohol abusers in *MIR29B2CHG*. This resulted in a mean predicted age of 1.4 years higher compared to the controls and this trend increased in older individuals. The association of alcohol abuse with epigenetic age acceleration, as determined by the prediction analysis performed based on *MIR29B2CHG*, was small but significant (β = 0.190; *P-*value = 0.007). However, the observed alteration in DNA methylation of *MIR29B2CHG* had a non-significant effect on age estimation with the VISAGE age prediction model. The mean absolute error in the alcohol-abusing cohort was 3.1 years, compared to 3.3 years in the control group. At the same time, upregulation of *MIR29B2CHG* expression may have a biological function, which merits further studies.

**Supplementary Information:**

The online version contains supplementary material available at 10.1007/s00414-021-02665-1.

## Introduction

DNA methylation (DNAm) is an epigenetic modification involved in the regulation of gene expression and processes responsible for normal organism development and growth, including genome imprinting and X chromosome inactivation. An important finding in DNA methylation (DNAm) research was the demonstration that the alteration of DNAm patterns is correlated with age [1–3]. As a result of this discovery, epigenetic clocks have been developed that provide an accurate estimate of chronological age [4–6]. A number of subsequent studies have shown that these epigenetic clocks are able to capture epigenetic age acceleration (EAA), observed when an individual’s DNA methylation age is greater than their chronological age [7–9]. EAA has been documented in aging-related diseases, stress, cancer, and cardiovascular disease, and is a predictor of mortality [10–14]. In addition, it was found that DNA methylation levels at some loci may be more sensitive to an individual’s overall health condition [12], and further studies developed the dedicated EAA-capturing clocks [8, 9]. The ability to measure biological age and EAA has practical implications including the prevention and treatment of diseases and life extension [15, 16]. In contrast, current forensic DNA analysis uses age estimation as a source of investigative leads and aims to accurately predict chronological age, treating the difference between predicted and actual ages as measurement error. EAA, which causes an increase in the chronological age prediction error, shows some level of heritability but can also be modified by extrinsic factors such as clinical and lifestyle parameters [10, 17–19]. As sensitivity to environmental influence can vary between markers, the appropriate selection of stable forensic age predictors can enable a more accurate estimation of chronological age [20, 21]. Recently, the VISAGE enhanced tool for age estimation of DNA from somatic tissues including blood, buccal cells, and bones (hereafter referred to as VISAGE Age Tool) was developed by the VISAGE (VISible Attributes through GEnomics) Consortium [22]. The VISAGE Age Tool targets eight well-validated DNA methylation markers (*ELOVL2, MIR29B2CHG*, *KLF14*, *FHL2*, *TRIM59*, *PDE4C*, *EDARADD*, and *ASPA*), of which 6 CpG sites are included in the age estimation model for blood. These are *ELOVL2* C7 chr6:11,044,634, *MIR29B2CHG* C1 chr1:207,823,681, *KLF14* C4 chr7:130,734,375, *FHL2* C1 chr2:105,399,282, *TRIM59* C8 chr3:160,450,202, and *PDE4C* C5 chr19:18,233,105. The blood model explains 98.2% of the age-related variance and predicts age with a mean absolute error (MAE) of 3.2 years [22]. The usefulness of this tool in forensics is particularly due to the high sensitivity of DNA methylation measurements using multiplexed targeted massively parallel sequencing (MPS), which provides good accuracy of chronological age prediction. Validation of this method should include an assessment of the effect of potential confounders on age estimation. It has been shown that alcohol dependence leads to premature aging and precipitates the onset of age-related diseases [23–25]. Excessive alcohol consumption has been hypothesized to reduce telomere length, partly due to oxidative stress related to acetaldehyde accumulation in the body [26–28]. There are few studies that have explored the influence of alcohol consumption on epigenetic age, and their results are inconclusive. The use of Horvath’s epigenetic clock detected increased age acceleration during the childhood and adolescence of the offspring of drinkers [29]. Furthermore, studies exploring the aging rate in relation to alcohol consumption using Hannum’s epigenetic clock reported a dose-dependent influence with accelerated aging in light and heavy drinkers but decelerated aging in moderate alcohol drinkers [30]. A positive influence of moderate doses of alcohol consumption on age was also reported by Quach et al. [31], who considered that this may be related to the anti-inflammatory effects of light alcohol intake, which are associated with decreased circulating levels of inflammatory markers such as IL-6 and CRP [31, 32]. The PhenoAge clock indicated epigenetic age acceleration in alcohol use disorder and suggests that disease severity further accelerates epigenetic aging [33]. Considering that excessive alcohol abusers may have different levels of methylation compared to healthy controls, we examined whether severe alcohol consumption might be associated with accelerated aging and increased error of age estimation using the VISAGE Age Tool. Our objective was to track potential dysregulation of DNA methylation in blood through excessive alcohol abuse at the eight markers used for forensic chronological age estimation.

## Materials and methods

### Study samples

The study was approved by the ethics committee of the Jagiellonian University in Krakow (KBET/122.6120.86.2017). Samples were collected during routine autopsies, performed by a forensic medical examiner at the Department of Forensic Medicine, Jagiellonian University Medical College in Krakow, Poland. The time from death to autopsy ranged from 1 to 5 days. Blood was collected from 212 deceased people aged 30–60 at the time of death, including 106 individuals extensively abusing alcohol and 106 sex- and age-matched controls. Extensive alcohol abusers were identified based on family/community history, medical records, and prosecutor’s documentation. While analyzing the medical data, we focused on the following underlying causes: long-term abuse of alcohol (at least several years), alcoholic liver disease, alcohol dependence syndrome leading to hospital treatment, acute alcohol poisoning, and incidents of alcohol withdrawal seizures, usually characterized by one or two generalized tonic–clonic events, although sometimes status epilepticus was reported. The following information on the study participants was obtained from medical examination reports and autopsies. The standard forensic autopsy protocol includes an investigation into alcohol addiction and we were able to focus on the significant pathological changes associated with alcohol abuse and thus direct information about alcohol-induced damage to the body. The chronic abuse of alcohol often leads to specific pathologic changes that affect organs such as the digestive and cardiovascular systems. In the digestive system, the liver may exhibit steatosis (fatty liver), steatohepatitis (alcoholic hepatitis), or cirrhosis, which was considered for classification. In addition, an important selection factor was the histopathological assessment of soft tissue changes characteristic of alcohol abusers. Given the range of alcohol-related pathologies, particular attention was paid to the liver (focal/diffuse steatosis, fibrosis, cirrhosis), pancreas (acute and chronic pancreatitis), lung (pneumonia), and heart (cardiomyopathy). The alcohol-related pathologies were determined from a combination of macroscopic examination of organs during the autopsy and histological assessments. Furthermore, toxicological testing at the time of death (drug and /or alcohol use) was made in both tested groups. In all alcohol abusers, the whole blood ethanol concentration was high, unlike the control group. There was no organ damage or pathological changes noticed in the control group, according to autopsy and histopathology protocols, and no alcohol abuse was reported according to the family/community interview, medical records, and prosecutor’s documentation. In both tested groups, the psychiatric disorders or abuse of narcotic drugs or psychotropic substances was not recorded.

### DNA methylation analysis

Peripheral blood (500 μL) was collected on NucleoCard (Macherey–Nagel, Düren, Germany), allowed to dry overnight at room temperature, and stored under such conditions until analysis. Total DNA was extracted from the card punches using a silica-based method with the Sherlock AX kit (A&A Biotechnology, Gdynia, Poland), according to the manufacturer’s guidelines. DNA was quantified using the Qubit dsDNA HS Assay Kit on a Qubit 4 Fluorometer (Thermo Fisher Scientific, Waltham, MA, USA), following manufacturer’s guidelines. Bisulfite conversion (BC) was performed with 500 ng DNA using the EZ DNA Methylation-Direct Kit (Zymo Research, Irvine, CA, USA) and eluted in 25 μL. DNA methylation levels were quantified using the VISAGE Age Tool [22]. The VISAGE Age Tool MPS assay is based on PCR enrichment of targeted regions from bisulfite-converted DNA [34] and allows analysis of 44 CpG sites in eight age informative markers, namely *ELOVL2*, *KLF14*, *TRIM59*, *FHL2*, *MIR29B2CHG*, *PDE4C*, *ASPA*, and *EDARADD*. In brief, 10 μL of the bisulfite-converted DNA samples were amplified in one multiplex PCR assay and libraries were prepared using the KAPA Hyper Prep Kit and KAPA Unique-Dual Indexed Adapters (both Roche, Basel, Switzerland). Four out of 212 samples were excluded from sequencing due to low library concentrations and therefore a set of 208 samples was sequenced on the MiSeq FGx instrument using the MiSeq FGx Reagent Kit (600 cycles; both Verogen, San Diego, CA, USA) with 2x 200 cycles. Control and corresponding test samples were sequenced together in one run if the used indexes allowed for multiplexing to avoid batch effects. Pooled libraries were diluted to 7 pM and sequenced with a 2 μL 20 pM PhiX spike in. Generated FASTQ files were aligned against a custom reference using bwa-meth as described in Woźniak et al. [22]. The number of reads at all 44 CpG positions were extracted using bam-readcount with minimum mapping quality and minimum base quality set to 30 (https://github.com/genome/bam-readcount). DNA methylation levels were calculated as the C reads percentage (C reads/(C reads + T reads) * 100). For two samples, the established minimum threshold of 1000 paired (R1 + R2) reads was not reached (within CpGs in *PDE4C* and *ELOVL2*), which resulted in a final missing data rate of only 0.25%.

### Statistical analyses

The DNA methylation percentage at particular CpG sites was compared between individuals from the alcohol abusers’ group and sex- and age-matched healthy controls using an independent sample Student’s *t* test. A proper distribution of age in the alcohol abusers’ group and controls was confirmed with a nonparametric Kolmogorov–Smirnov test. The sex ratio between the tested groups was compared with chi-square (*χ*^2^) statistics. Due to the known differences in age prediction accuracy between younger and older individuals [35], the whole cohort was divided into two age categories including individuals aged 30–45 and 46–60 years, and calculations were performed for each age group separately. Age predictions were made using a linear regression-based age prediction model for blood developed in Woźniak et al. [22]. The model was developed based on individuals with no signs of alcohol abuse. This model comprises 6 CpG sites in 6 genes, namely *ELOVL2*, *MIR29B2CHG*, *KLF14*, *FHL2*, *TRIM59*, and *PDE4C*. In addition, a model based on *MIR29B2CHG* alone was developed using linear regression with enter mode of variable selection. Samples lacking data for *ELOVL2* and *PDE4C* were excluded from all prediction analyses. For all missing samples, sex- and age-matched samples were excluded from statistical analysis resulting in a group of 200 individuals analyzed using the VISAGE enhanced age model for blood and the *MIR29B2CHG* C1 model. The predicted age of alcohol-abusing individuals was compared with their true chronological age to calculate MAE. An independent sample Student’s *t* test was used to compare mean predicted age and MAE designated for the tested groups. Epigenetic age acceleration (EAA) was designated in the form of residuals calculated from linear regression analysis, where predicted age was treated as the dependent variable and chronological age as the independent variable as described in [7, 36]. Association analysis of alcohol abuse with EAA was tested using linear regression, controlling the results for the effects of age and sex. Analyses were performed using IBM SPSS Statistics 26 and R [37].

## Results

### Assay performance and sequence quality

The VISAGE Age Tool showed a good overall performance throughout the four sequencing runs, with high sample coverage (mean = 316,048.3 ± 28,299.9 paired reads) and high read depth at all 44 target CpG positions (mean = 39,423.5 ± 3553.6 paired reads; Supplementary Fig. 1a). Only two of 208 samples showed a drop below the lower limit of 1000 paired reads at *PDE4C* or *ELOVL2* positions and were consequently excluded from analysis along with the matching samples. Normalized read depth was calculated from one CpG position per marker to assess read distribution across the eight amplicons (Supplementary Fig. 1b). The observed read distribution shows two overperforming markers (*TRIM59* and *FHL2*) leading to a lower-than-expected amount of reads for *ASPA*, *ELOVL2*, *KLF14*, *EDARADD*, and *PDE4C*. However, the average read depth at these CpG positions was still above the threshold set for quantitative DNAm analysis, showing a minimum mean read depth per marker of 9764.6 ± 1100.7 paired reads at *PDE4C*. Results were stable throughout all runs indicating a robust performance of the assay. Sequence quality was assessed by calculating the base misincorporation rates at target CpG positions showing a mean of 0.1% for all four runs. Successful bisulfite conversion was controlled by calculating the average percentage of T reads at all non-CpG C reads of the eight amplicons and showed an overall mean conversion efficiency of 99.6% per sample (minimum = 99.2%).

### Differences in DNA methylation at CpG sites

**Fig. 1:**
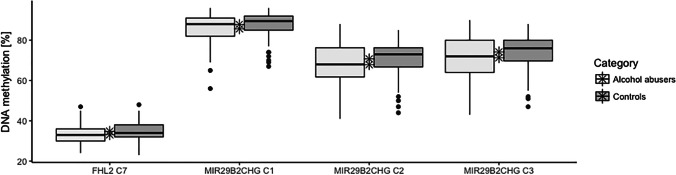
Altered DNA methylation at *MIR29B2CHG* C1, C2, C3*;* and *FHL2* C7. The mean DNA methylation values are marked with an asterisk

The alcohol abusers group used in statistical analyses consisted of 100 individuals with a mean age of 46.19 ± 8.46 (min = 30; max = 60) and included 83 males (83%). The group of controls consisted of 100 individuals at a mean age of 46.19 ± 8.46 (min = 30; max = 60), likewise including 83 males (83%). The groups of alcohol abusers and controls were perfectly matched by age and sex. Univariate association analysis involved 44 CpG sites and was conducted separately for alcohol abusers and controls for each CpG (Supplementary Table 1). In brief, significant association with age was observed for all CpGs in the group of controls and for all CpGs, except *EDARADD* (C2 chr1:236,394,371), in the alcohol abusers’ group (*P*-value = 0.476). However, another CpG site in *EDARADD* (C1 chr1:236,394,383) showed significant association with age in both tested groups (alcohol abusers with *P*-value = 0.003 and controls with *P*-value = 8 × 10^−6^). Mean values of DNA methylation were compared between both groups using an independent sample Student’s *t* test (Supplementary Table 2). Significantly altered DNA methylation was noted in three CpGs in *MIR29B2CHG* (C1, C2, C3 with *P*-value of 0.029, 0.033, and 0.025, respectively). A difference in DNAm between alcohol abusers and controls was also observed in one CpG in *FHL2* (C7 with *P*-value = 0.014). For all four of these CpGs, DNA methylation was found to be lower in alcohol abusers than in controls (Fig. 1).

The *MIR29B2CHG* C1 site is included in the VISAGE Age Tool for blood, and thus, the altered DNA methylation values at this site may influence age predictions [22]. The mean difference in obtained DNA methylation levels comprised 2.1, 2.8, and 3.0 for C1, C2, and C3 in *MIR29B2CHG*, respectively, and 1.7 for C7 in *FHL2*. As DNA methylation decreases with age in the case of *MIR29B2CHG*, this suggests a slightly faster aging of alcohol abusers. The alteration observed in *FHL2* C7 is inconsistent with the DNA methylation patterns in the other nine cytosine sites studied at this locus. Since *FHL2* is a locus where DNA methylation increases with age, this indicates slower aging in alcohol abusers. However, the *FHL2* C7 site is not included in any of the models and thus has no effect on age prediction.

### Differences in the predicted age

In the next step, age was predicted for all samples using the VISAGE enhanced model for blood. Supplementary Fig. 2 shows the DNA methylation level for 6 CpG sites included in the VISAGE enhanced model. Mean absolute error (MAE), mean error (ME), and predicted age were compared between alcohol abusers and controls. The MAE for alcohol abusers was 3.1 and for controls 3.3, but the difference was not statistically significant (*P*-value = 0.582; Table 1). The mean predicted age of alcohol abusers was 1.4 years higher compared to controls; however, this difference was not statistically significant (*P*-value = 0.311).

**Table 1:** Age prediction parameters for alcohol abusers (*N* = 100) and controls (*N* = 100). Predictions made using the VISAGE enhanced model for blood

Compared groups	*N*		Std. deviation	Mean difference	Std. error difference	*P*-value
		MAE				
**Alcohol abusers**	100	3.099	2.894	− 0.229	0.416	0.582
**Controls**	100	3.329	2.985			
		ME				
**Alcohol abusers**	100	0.972	4.138	1.419	0.608	0.021
**Controls**	100	− 0.447	4.461			
		Mean predicted age				
**Alcohol abusers**	100	47.162	9.970	1.419	1.396	0.311
**Controls**	100	45.743	9.774			

A similar trend was noted when the subjects were divided into two age categories. The mean predicted age of alcohol abusers was about 2 years higher when compared to controls in the category of older individuals (aged > 45 years, *P*-value = 0.133). The mean error was 1.9 years higher in elderly individuals and this difference was significant (*P*-value = 0.033). The results are presented in Table 2.

**Table 2:** Age prediction parameters for alcohol abusers and controls included in two age categories: 1: 30–45 years old and 2: 46–60 years old

**a.** **Mean absolute error (MAE) in two age categories**						
Compared groups	*N*	MAE	Std. deviation	Mean difference	Std. error difference	*P*-value
Age category 1						
Alcohol abusers	47	2.556	2.158	− 0.927	0.559	0.101
Controls	47	3.483	3.167			
Age category 2						
Alcohol abusers	53	3.582	3.365	0.390	0.604	0.520
Controls	53	3.192	2.836			
**b.** **Mean error (ME) in two age categories**						
Compared groups	*N*	ME	Std. deviation	Mean difference	Std. error difference	*P*-value
Age category 1						
Alcohol abusers	47	0.373	3.345	0.897	0.842	0.289
Controls	47	− 0.524	4.706			
Age category 2						
Alcohol abusers	53	1.503	4.700	1.882	0.873	0.033
Controls	53	− 0.379	4.276			
**c.** **Mean predicted age in two age categories**						
Compared groups	*N*	Mean predicted age	Std. deviation	Mean difference	Std. error difference	*P*-value
Age category 1						
Alcohol abusers	47	38.778	5.716	0.897	1.241	0.472
Controls	47	37.880	6.302			
Age category 2						
Alcohol abusers	53	54.598	6.362	1.882	1.242	0.133
Controls	53	52.715	6.428			

**Fig. 2:**
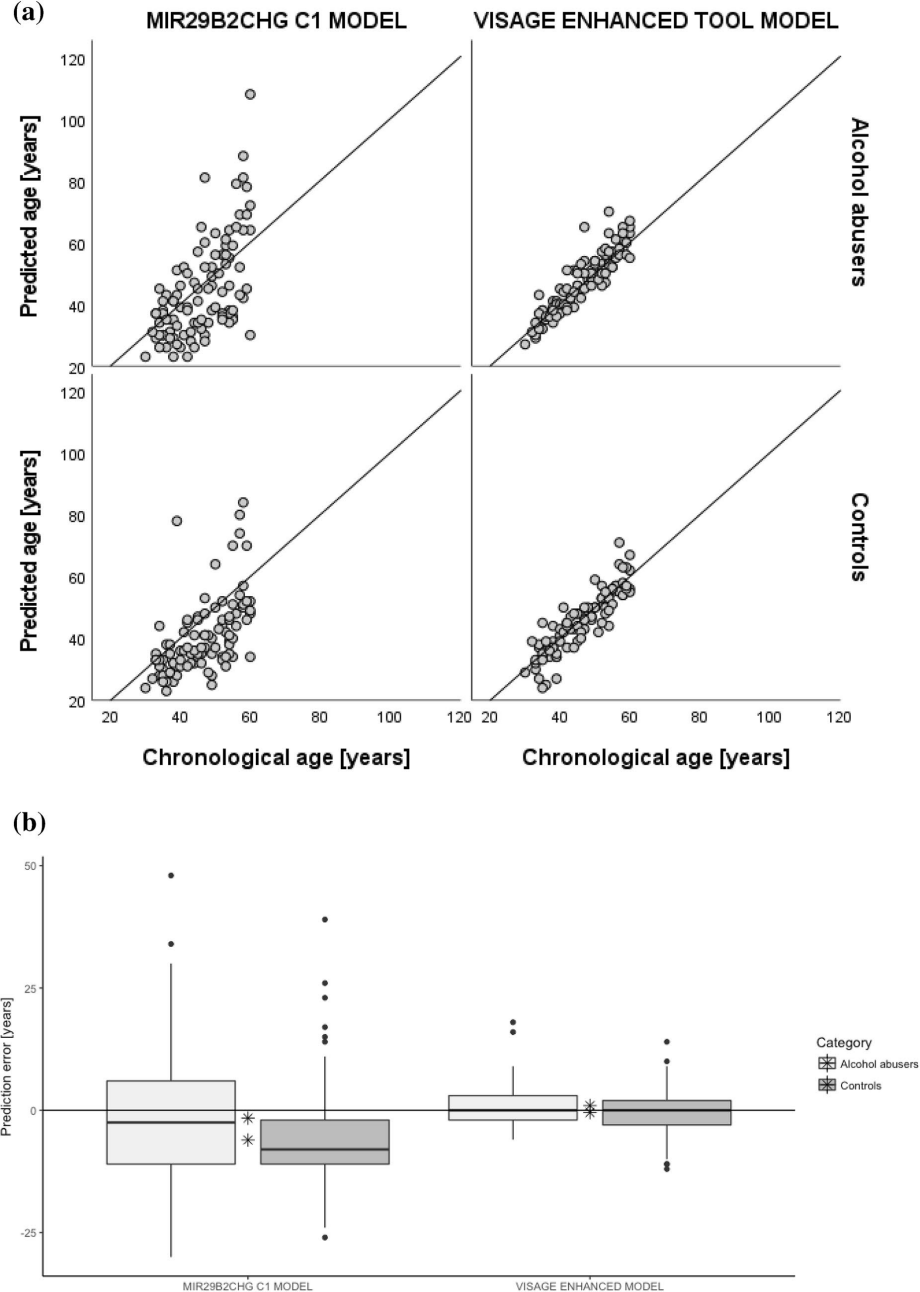
Age prediction parameters in alcohol abusers and controls using the model based on *MIR29B2CHG* C1 alone and the VISAGE age model. **a** Predicted age. **b** Prediction error. The mean error is marked with an asterisk. The horizontal line shows the error value equal to 0

Out of six CpGs included in the age prediction model for blood, significant differences in the percentage of DNA methylation were found only in *MIR29B2CHG* C1. Thus, the data generated in Woźniak et al. [22] was used to develop a model that included only *MIR29B2CHG* C1. Based on this model, MAE for alcohol abusers was 10.1 and for controls 9.6, and the difference was not statistically significant (*P*-value = 0.634). The mean predicted age of alcohol abusers was 4.5 years higher, compared to controls, and the difference was statistically significant (*P*-value = 0.029). When the mean error was compared, significant differences were observed—the ME was 4.5 years higher in alcohol abusers, when compared to the controls (*P*-value = 0.007). The results are presented in Fig. 2 and Table 3.

**Table 3:** Age prediction parameters for alcohol abusers (*N* = 100) and controls (*N* = 100). Predictions made using a model based on *MIR29B2CHG* C1

Compared groups	*N*		Std. deviation	Mean difference	Std. error difference	*P*-value
		MAE				
**Alcohol abusers**	100	10.084	8.027	0.503	1.054	0.634
**Controls**	100	9.581	6.835			
		ME				
**Alcohol abusers**	100	− 1.604	12.828	4.453	1.634	0.007
**Controls**	100	− 6.056	10.119			
		Mean predicted age				
**Alcohol abusers**	100	44.586	16.060	4.453	2.025	0.029
**Controls**	100	40.134	12.338			

Additionally, we predicted the age in different age groups for the *MIR29B2CHG* C1 model. The predicted age of younger alcohol abusers (≤ 45) was 1.4 years higher, compared to age-correlated controls, but the difference was not statistically significant (*P*-value = 0.426). In the group of elderly alcohol abusers, the difference was up to 7.2 years (i.e., alcoholics are predicted to be 7.2 years older than controls on average) and this was a statistically significant result (*P*-value = 0.017). Finally, we assessed EAA, which is a measure of the discrepancy between the biological age (i.e., DNA methylation age) and the chronological age. Individuals with positive age acceleration values (i.e., the biological age being greater than their chronological age) are experiencing accelerated aging. Our study showed that alcohol abuse is significantly correlated with EAA, both when using the original VISAGE age model (*P*-value = 0.020) and a model based only on *MIR29B2CHG* C1 (*P*-value = 0.007). However, when alcohol abusers were divided into two age groups, no accelerated aging was found in alcohol abusers aged 45 years or younger, while age acceleration occurred in those aged 46 years or older (*P*-value = 0.035). This was more significant when using a model based on *MIR29B2CHG* C1 (*P*-value = 0.009; Table 4).

**Table 4:** Epigenetic age acceleration calculated based on both predictive models in all samples and in two age categories: 1: 30–45 years old and 2: 46–60 years old

Predictive model	Groups compared	Age category	Epigenetic age acceleration		
			Effect size*	*t* statistic	*P*-value
**VISAGE blood**	Alcohol abusers vs. controls	All	0.164	2.350	0.020
***MIR29B2CHG* C1**	Alcohol abusers vs. controls	All	0.190	2.748	0.007
**VISAGE blood**	Alcohol abusers vs. controls	1	0.111	1.090	0.279
		2	0.207	2.142	0.035
***MIR29B2CHG* C1**	Alcohol abusers vs. controls	1	0.087	0.886	0.378
		2	0.252	2.674	0.009

^*^Effect sizes are the standardized beta coefficients from linear regression models adjusted for age (years) and sex

## Discussion

Forensic DNA phenotyping is an evolving discipline that uses genetic and epigenetic data to develop, optimize, and validate predictive tools and models for the purpose of establishing forensic intelligence. Currently, the most promising available predictive methods include inference of biogeographic ancestry, eye, hair and skin color prediction, and age estimation [38–41]. The VISAGE enhanced tool for age estimation from somatic cells contains eight carefully selected markers that can be analyzed in forensic samples and predict age in blood, buccal cells, and bones [22]. In this study, we investigated the stability of age prediction of this set of markers in blood of excessive alcohol abusers, which potentially represents a confounding factor.

The examination of the data using the VISAGE Age Tool for blood showed that the MAE for alcohol abusers and controls analyzed in our study was in line with the result obtained for the original testing set [22]. Notably, although statistical significance was not detected, the mean predicted age of alcohol abusers was higher compared to controls. This difference was due to alterations of DNA methylation in C1 in *MIR29B2CHG*. Considering the predictive power of the VISAGE enhanced model for blood, *MIR29B2CHG* is ranked in 3rd position after *ELOVL2* and *PDE4C*. In univariate analyses, C1 alone explained almost 81% of the age-related variation [22]. Increased hypomethylation of *MIR29B2CHG* C1 was found in alcohol abusers compared to controls, and this created an increase in the predicted age of individuals from this group. Association analysis of alcohol abuse with EAA showed a significant correlation and was most evident after dividing alcohol abusers into two age categories. Although there was no association of alcohol abuse with accelerated aging in the younger group, statistical significance was observed in older individuals, for both the original VISAGE enhanced age model and the adapted model based on *MIR29B2CHG* C1 alone. It can be hypothesized that prolonged periods of alcohol consumption will be responsible for the stronger effect of methylation loss observed in this marker.

The observed accelerated hypomethylation of the *MIR29B2CHG* promoter region in response to extensive alcohol consumption may result in upregulation of *MIR29B2CHG*. The gene *MIR29B2CHG* located on chromosome 1q32.2 is known to contain two microRNAs, Mir-29b2 and Mir-29c, and probably is a so-called host gene for these microRNAs [42]. Interestingly, an increased gene expression of Mir-29b and Mir-29c was found in a mouse model of Hutchinson–Gifford progeria syndrome [43]. Previous studies have shown limited alterations of DNA methylation levels in five markers included in both VISAGE age prediction tools [22, 44] in response to different types of external factors, including extreme exercises and various diseases, with no effect on *MIR29B2CHG* observed in these studies [36, 45]. However, this gene was hypermethylated in patients after hematopoietic stem cell transplantation (HSCT). It has been speculated that *MIR29B2CHG* can be involved in graft function after HSCT, impacting the self-renewal of hematopoietic stem cells [46]. The exact biological function of the observed differences in DNA methylation at the *MIR29B2CHG* gene promoter in response to excessive alcohol consumption is unclear. Tharakan et al. [42] hypothesized that methylation changes may reflect critical biological mechanisms in aging. The transcriptional repressor CTCF modulates a so-called hemimethylation of genomic DNA, which could cause a progressive loss of methylation at some loci over the course of cell division. Consistent with this idea, CTCF binding is prevalent at *MIR29B2CHG*, which shows decreased methylation during aging [42].

Chronic alcohol abuse can accelerate biological processes associated with aging, thus leading to an earlier onset of age-related diseases by activating the hypothalamic–pituitary–adrenal axis and increasing glucocorticoid levels [23]. Interestingly, by reducing the supply of S-adenosylmethionine, ethanol may decrease the expression of DNA methyltransferases and increase the expression of demethylase, thereby negatively regulating global DNA methylation [47]. Importantly, these ethanol-induced epigenetic changes persist well after ethanol or its metabolites have disappeared. Hence, even a transient ethanol exposure on tissues, organs, and organisms can be sustained across a wide range of timescales, with the potential to drive persistent gene regulatory changes underlying fetal alcohol spectrum disorders, cancer, and metabolic disorders [47–49].

We can speculate that the small age acceleration that we have captured studying blood of alcohol abusers may have a larger effect size in other tissues. Tissue-specific differences have been reported from the effects of alcohol consumption on an individual’s DNA methylation profile, e.g., between liver and brain [50] and in postmortem brain tissue, when DNA CpG islands showed both hyper- and hypomethylation [51]. Similarly, DNAm age was shown to be higher than chronological age in one blood dataset and one liver tissue dataset of individuals with alcohol dependence, but not in brain tissue. Interestingly, the average chronological age in blood samples was approximately 15 years younger than that of the postmortem brain tissue samples and liver cirrhosis samples [52].

Importantly, from the perspective of forensic genetics, the observed change in DNA methylation in *MIR29B2CHG* due to excessive alcohol abuse has a non-significant effect on epigenetic age prediction using the VISAGE enhanced age model. This is because the effect of alcohol on *MIR29B2CHG* is small and additionally, any changes are compensated by the other five predictors included in the model. The results obtained provide further evidence that the impact of environmental factors may have different meanings for individual differentially methylated regions, and a detailed analysis of molecular pathways involving specific age predictors regulated by external factors can provide insight into the functioning of the human genome. The study used data on alcohol abuse gathered from autopsy reports and family information. Therefore, some limitations need to be considered as no information was available on the amount and frequency of alcohol consumption, the family history of alcohol dependence, or drinking behavior. There was also a lack of data on environmental factors that can affect DNA methylation levels and epigenetic aging, such as diet or other lifestyle aspects. However, the study allowed for an overall assessment of the effect of objectively proven alcohol abuse, along with other factors associated with this extreme behavior, on the VISAGE Age Tool for epigenetic age prediction.

## Conclusions

In conclusion, the present study shows a low impact of excessive alcohol abuse on DNA methylation patterns in the eight age markers studied and, consequently, on the accuracy of epigenetic age prediction based on the six CpG model we have developed for blood. This confirms the high informativeness of the VISAGE Age Tool for epigenetic prediction of blood age in forensic analyses. Further studies on a potential biological function of the identified effect of alcohol abuse on DNA methylation in *MIR29B2CHG* may be interesting, as well as confirmation of this effect in other forensically relevant tissues or biological fluids. It will also be interesting to assess the influence of other potential confounders on epigenetic age prediction and age acceleration.

## Supplementary Information

Below is the link to the electronic supplementary material.

Supplementary file1 (PDF 198 KB)

Supplementary file2 (PDF 243 KB)

Supplementary file3 (XLSX 17 KB)

Supplementary file4 (XLSX 21 KB)

## Data Availability

All data are available at the Malopolska Centre of Biotechnology, Jagiellonian University, ul. Gronostajowa 7A, 30–348 Krakow, Poland, and can be requested from the authors.
