# Supplementary material for: Impact of excessive alcohol abuse on age prediction using the VISAGE enhanced tool for epigenetic age estimation in blood
Source: Int J Legal Med. 2021 Aug 18;135(6):2209–19. doi: 10.1007/s00414-021-02665-1 (PMC8523459; doi:10.1007/s00414-021-02665-1)
Supplement: Supplementary file 1 — Supplementary file1 (PDF 198 KB) [file 414_2021_2665_MOESM1_ESM.pdf]

Piniewska-Róg D, Heidegger A et al.: Impact of excessive alcohol abuse on age prediction using the VISAGE enhanced tool for epigenetic age estimation in blood

SUPPLEMENTARY MATERIAL

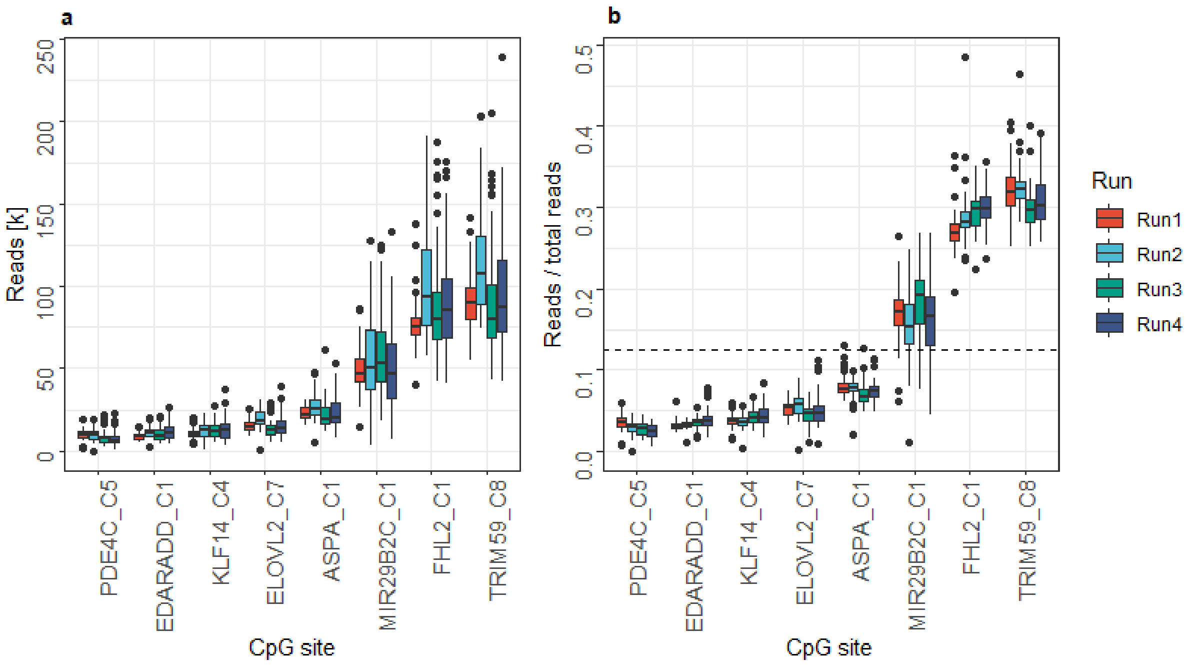

Supplementary Fig. 1 (**a**) Read depth at one CpG site per amplicon. (**b**) Normalized read depth (reads/total reads) of the respective eight CpG sites. The dashed line indicates the expected read distribution (one divided by the eight amplicons). Boxplots are colored by sequencing run (Run 1 & 2: N = 51, Run 3 & 4: N = 53).
