# Supplementary material for: Impact of excessive alcohol abuse on age prediction using the VISAGE enhanced tool for epigenetic age estimation in blood
Source: Int J Legal Med. 2021 Aug 18;135(6):2209–19. doi: 10.1007/s00414-021-02665-1 (PMC8523459; doi:10.1007/s00414-021-02665-1)
Supplement: Supplementary file 2 — Supplementary file2 (PDF 243 KB) [file 414_2021_2665_MOESM2_ESM.pdf]

Piniewska-Róg D, Heidegger A et al.: Impact of excessive alcohol abuse on age prediction using the VISAGE enhanced tool for epigenetic age estimation in blood

SUPPLEMENTARY MATERIAL

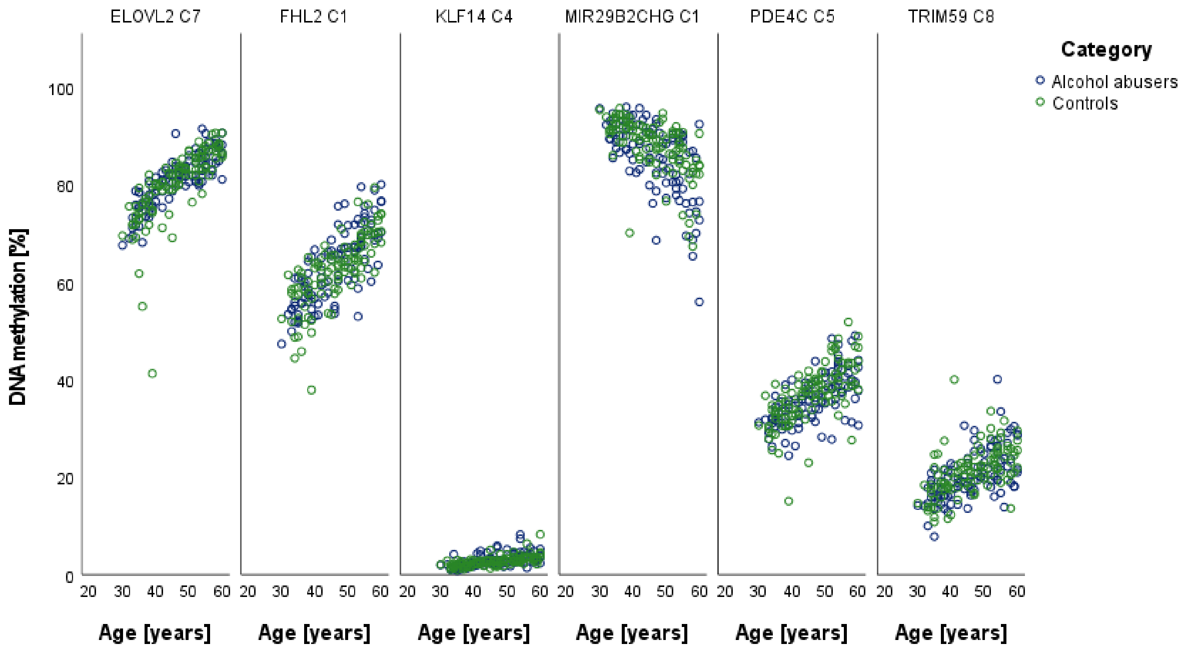

Supplementary Fig. 2 Scatter plots of the DNA methylation level as a function of chronological age for 6 CpG sites included in the VISAGE enhanced model.
